# Enablers and barriers to COVID-19 vaccine uptake in an urban slum in Lagos, Nigeria: informing vaccine engagement strategies for the marginalized

**DOI:** 10.1093/inthealth/ihad009

**Published:** 2023-02-17

**Authors:** Obianuju B Ozoh, Ayesha O Akinkugbe, Morayo A Olukoya, Ifedayo M O Adetifa

**Affiliations:** Department of Medicine, College of Medicine, University of Lagos and Lagos University Teaching Hospital, Idi-Araba, Lagos; Department of Medicine, College of Medicine, University of Lagos and Lagos University Teaching Hospital, Idi-Araba, Lagos; Lagos State Primary Health Care Board, Lagos, Nigeria; Nigeria Centre for Disease Control, Abuja, Nigeria; Department of Paediatrics and Child Health, College of Medicine, University of Lagos, Lagos, Nigeria

**Keywords:** acceptance, barriers, COVID-19, Nigeria, urban slums, vaccine

## Abstract

**Background:**

Vaccination against coronavirus disease 2019 (COVID-19) is a cost-effective mitigation strategy against the pandemic. As the COVID-19 vaccine becomes more available, low uptake is now a global threat and understanding the underpinnings in local contexts is a priority for intervention development. We aimed to evaluate behavioural determinants of COVID-19 vaccine acceptance that could inform engagement strategies to improve vaccine uptake in Makoko, an urban slum in Lagos, Nigeria.

**Methods:**

A population-based case–control study utilized the barrier analysis (BA) approach to evaluate the beliefs and behaviours of 45 ‘doers’ and 45 ‘non-doers’. The standardized BA tabulation sheet was used to assess differences in the proportions between the two groups to identify significant factors that could be addressed through a behaviour change strategy.

**Results:**

Perceived social norms (family, friend, healthcare workers) that approve the vaccine and expected vaccine protection against diseases among doers were determinants of behaviour. Perceived poor accessibility, safety concerns, lack of trust, low vaccine efficacy and low susceptibility to the infection were the most important determinants of behaviour among non-doers.

**Conclusions:**

Measures to improve COVID-19 vaccine acceptance in Makoko should include improvement in accessibility and exposing myths and misinformation through clear, concise and evidence-based community education delivered by trusted persons such as healthcare workers and religious leaders.

## Background

Vaccination against severe acute respiratory syndrome coronavirus 2 (SARS-CoV-2) is the most cost-effective mitigation strategy against the ongoing coronavirus disease 2019 (COVID-19) pandemic.^1^ The currently observed disconnect between COVID-19 case numbers and adverse outcomes (severe disease, hospitalization and deaths) in highly vaccinated populations highlights the opportunity vaccines offer if the world is to emerge from this pandemic.

Of the nearly 10 billion doses of COVID-19 vaccines administered globally, only about 10% have been administered in low-income countries, showcasing the failure of global health solidarity to ensure equitable access to vaccines in low- and middle-income countries (LMICs).^2^ While the interventions by the World Health Organization (WHO), the World Bank and other global partners through COVID-19 Vaccines Global Access (COVAX) facilities and the African Vaccine Acquisition Trust (AVAT) have moved the needle, most LMICs may not meet the WHO 70% population coverage by mid-2022.^3^

Beyond the hurdle of equitable vaccine access, there are significant pockets of vaccine-hesitant populations, even in highly vaccinated high-income countries. Vaccine hesitancy refers to a delay in acceptance or refusal of vaccination despite the availability of vaccination services.^4^ Global reports of COVID-19 vaccine hesitancy indicate rates as high as 70%.^5^ Contributory factors range from traditional concerns (low perceived risk of disease, safety concerns, religious and cultural preferences) to those unique to COVID-19 vaccines (political factors and concerns related to the rapidity of development and conspiracy theories).^5^

Given Nigeria's status as the most populated African country, its COVID-19 vaccination coverage will have a substantial impact on subregional and regional targets. Only 5.5% of Nigerians had received one dose of the COVID-19 vaccine and 2.3% were fully vaccinated (defined as two doses of vaccine) at the end of January 2022, despite availability to all adults free of charge. The COVID-19 vaccine is administered free of charge at primary healthcare centres in all local government areas (n=774) nationwide, including the health centre that serves the Makoko community. It is essential to gain an understanding of the factors associated with this low coverage (0.03/100 population) of vaccination.^2^ Vaccine hesitancy appears to be an important contributing factor since more than half of adult Nigerians are unwilling to receive the COVID-19 vaccine even with the ongoing mass vaccination campaign.^6^ Understanding the factors that drive low vaccine uptake can help inform interventions to improve uptake, which will reduce transmission, including the emergence of new variants.

Lagos, the capital city of Lagos State, has been the epicentre of COVID-19 in Nigeria through all four waves of the pandemic, yet only 18% of its population have received a dose of COVID-19 vaccine and just 11% have received two doses.^7^ Typical of a rapidly expanding metropolis/megacity, Lagos is also home to >200 urban slums, with a teeming population residing in poor living conditions with social, infrastructure and economic deprivation, which are impediments to adherence to public health safety measures for preventing COVID-19.^8^ Populations in urban slums are underserved, with poor access to essential health services and even poorer health-seeking behaviour. They also typically have lower vaccination coverage for routinely delivered vaccines and are often left behind during mass vaccination campaigns due to low awareness of the value of vaccination and difficulty accessing services.^9^

Makoko is one of the most popular urban slums and it has received global recognition as the world's largest floating slum, or the ‘Venice of Africa’. Makoko and other slums in Lagos have suffered many decades of neglect in the developmental schema, with very poor living conditions, limited access to healthcare and health information being well-known challenges. In this study we assessed the behavioural determinants of COVID-19 vaccine acceptance among adults living in Makoko to identify areas of intervention and/or engagement to improve COVID-19 vaccine uptake in this and similar urban slums.

## Methods

### Study setting

Makoko, a slum community in the megacity of Lagos, is situated within Yaba, a suburb located in Lagos Mainland. It is an informal waterfront settlement with some homes built on stilts in the lagoon and extending into the Aderupoko and Salami-Baiyewunmi wards of the Yaba Local Community Development Association (LCDA). These extensions include swampy and dry land. It has a population of about 100 000 inhabitants who originally migrated from Badagry and the Republic of Benin. The community is predominantly Egun and Ilaje, in addition to Yoruba, Ibo and other ethnic groups. Migrants from other coastal communities of the Niger Delta, Benin, Ghana and Togo can also be found among the Makoko population.^10^

Transportation within the floating section is by boat (personal or commercial) and this limits the mobility of the inhabitants. Sanitation facilities are non-existent in the floating section and the water is polluted with human and animal waste. The situation in the land areas is not very different. The only healthcare facility for the community is located on land in the Aiyetoro area of Ori Oke/Makoko community. There is no facility for COVID-19 testing within the community, but vaccination for COVID-19 is available at the health centre located on land at the Yaba LCDA free of charge to all adults ≥18 y of age.

### Study design

The Barrier analysis (BA) approach uses a case–control study design to explore participants’ beliefs about a behaviour (in this case, receiving COVID-19 vaccination) and the most likely determinants of the behaviour. BA is a well-recognized formative approach for rapid assessment and understanding of behavioural determinants that can guide the timely development of strategies and messaging for behavioural change.^11^ The BA compares the responses of those who have adopted or plan to adopt a behaviour (‘doers’) with those who have not or who do not plan to adopt the behaviour (‘non-doers’). Understanding these differences has been found to inform the development of messaging and activities that could lead to adoption of the desired behaviour and has been considered in some instances superior to surveys and focus group discussions. The BA approach has been used in previous studies to understand determinants of behaviours including uptake of the COVID-19 vaccine.^12,13^ BA focuses on eight determinants of behaviour: perceived susceptibility, perceived severity, perceived action efficacy, perceived social acceptability, perceived self-efficacy, cues for action, perception of divine will and positive and negative attributes of the behaviour.

### Study population

Consenting adults ≥18 y of age who have resided in the Makoko community for a minimum of 1 year and who are aware of COVID-19 were included. Participants were recruited equally from Makoko and its extensions, comprising of the sites on the lagoon, the swampy area and dry land (Makoko, Aderupoko and Salami-Baiyewunmi).

### Sample size and sampling approach

To assess behaviour using the BA approach, a minimum sample size of 45 acceptors/doers and 45 non-acceptors/non-doers (determined by initial screening) is recommended.^11^ Comparison of the responses of 45 doers and 45 non-doers has been calculated to detect a statistically significant minimally relevant difference in the odds ratio (OR) of ≥3.0 with 95% confidence and a power of 80%.

Participants were equally recruited from randomly selected households within the three zones of the community using a door-to-door approach. They were approached in their homes and first screened to assess eligibility, based on the inclusion criteria: awareness of COVID-19 as a disease and awareness of the availability of a vaccine against the disease. Further categorization as doers was based on willingness/plan to receive or have already received the COVID-19 vaccine, while non-doers was based on unwillingness to receive the vaccine. We recruited one consenting adult who met the eligibility criteria per household. We continued recruitment until 45 doers and 45 non-doers were recruited.

### Data collection

Data collection was conducted by trained interviewers between 26 October and 7 November 2021. Each interviewer collected pilot data from three adults in the community as part of the training process. We used the responses from the questionnaires and the experiences of the interviewers to modify the questions for clarity and ensure they were asked in a consistent manner. The interviewers had training prior to study commencement and a supervisor was present in the community to ensure data integrity. The interviewers utilized an adaptation of the BA questionnaire that was used for a similar COVID-19 vaccine uptake study in an urban community in Bangladesh, a similar high-context community in an LMIC.^13^ Similar to the study in Bangladesh, we used the questionnaire to obtain participants’ sociodemographic information and beliefs regarding behavioural determinants of the COVID-19 vaccine by focusing on the most actionable points using the questions listed in Table 1.^13^ The study in Bangladesh was conducted at a time when COVID-19 vaccines had just become available to selected groups in the population. We modified the statement to reflect the current situation in Nigeria, where the vaccines are available to all adults ≥18 y of age. We framed the questions based on ‘now that the COVID-19 vaccines are available to you free of charge’, since the vaccine has been available to all adults for up to 1 y in Nigeria. We also obtained information on self-reported vaccination status (received at least one dose of the vaccine) among the doers.

**Table 1.** Description of the behavioural determinants assessed in this study

Name of determinant	Generic description	Contextualized questions
Perceived self-efficacy	An individual's belief that he/she can do a particular behaviour given his/her current knowledge, resources and skills	• What might make it easier and what might make it difficult to receive the COVID-19 vaccine now it is available to them free of charge?
Perceived social norms	The perception that people important to an individual think that he/she should do the behaviour (injunctive norms) and plan to do the behaviour (descriptive norms)	• What proportion of the people they know would get a COVID-19 vaccine now it is available to them free of charge? • Would close family and friends want them to get a COVID-19 vaccine? • Would their community and religious leaders want them to get a COVID-19 vaccine? • Who would approve of them getting a COVID-19 vaccine? • Who would disapprove of COVID-19 vaccination? • Would they get a COVID-19 vaccine if a doctor or nurse recommended it?
Perceived positive consequences	What positive things a person thinks will happen as a result of performing a behaviour	• What are the advantages of getting a COVID-19 vaccine?
Perceived negative consequences	The negative things a person thinks will happen as a result of performing a behaviour	• What are the disadvantages of getting a COVID-19 vaccine?
Access	The degree of availability (to a particular audience) of the needed facilities, services, or materials required to adopt a given behaviour.	• How difficult would it be to get to the clinic where vaccines are normally offered?
Cues to action reminders	The presence of reminders that help a person remember to do a particular behaviour	Not assessed (not likely to be relevant)
Perceived susceptibility/risk	A person's perception of how vulnerable or at risk they feel vis-à-vis the problem or disease	• What proportion of people in their community have had COVID-19? • How likely they thought it was that someone in their household would contract COVID-19? • How concerned they were about getting COVID-19?
Perceived severity	Belief that the problem or disease (which the behavior can prevent) is serious	• How serious would it be if someone who lives in their household contracted COVID-19?
Perceived action efficacy	The belief that by practicing the behaviour one will avoid the problem or disease; that the behaviour is effective in preventing the problem or disease	• If they were to get the COVID-19 vaccine, how likely would it be that they would get COVID-19 disease after that?
Perceived divine will	A person's belief that it is God's or the gods’ will for him/her to have the problem and/or to overcome it	• Does God approve or disapprove of people getting a COVID-19 vaccine? • They were also asked if they agreed with the statement, ‘Whether I get COVID-19 or not is purely a matter of God's will or chance. The actions I take will have little bearing on whether or not I get COVID-19’.
Policy	Laws and regulations (local, regional or national) that affect adoption of the behaviours and access to products and services	Not included, as the vaccination is ongoing and currently there are no mandates
Culture	The history, customs, lifestyles, values and practices within a self-defined group	Are there any cultural or religious reasons why they would not get a COVID-19 vaccine and, if yes, what are those reasons?

### Data analysis

Using the inductive and deductive approaches, the responses to the open-ended questions (qualitative data) in the questionnaire were coded to identify themes. The frequency of doers and non-doers who contributed to each identified theme was summed and presented as numerical data as recommended for the BA approach using the standard tabulation sheet.^14^ The frequency of the closed-ended questions was also summed for doers and non-doers. These frequencies were then imputed into the BA tabulation sheet, a standardized Excel tabulation sheet (Microsoft, Redmond, WA, USA) produced for analysis of the findings from a BA study based on the power calculation for a minimum sample size of 45 per group.^11^ Once the frequencies are imputed it assesses the differences in the proportion of responses between the doers and non-doers and computes the OR.^11^ We set the level of significance at p<0.05 and factors that were statistically significant were identified to represent targets that could be addressed through a behaviour change strategy.

### Ethical considerations

Ethical approval was obtained from the Health Research Ethics Committee of the Lagos University Teaching Hospital, Lagos, Nigeria. Informed written consent was obtained from all participants and all data were de-identified and confidentiality was assured.

## Results

We screened 126 adults to reach the required sample size of 45 doers and 45 non-doers needed for the BA analysis.

### Sociodemographic characteristics of participants

Table 2 describes the sociodemographic characteristics of the 90 participants. There were four females. Males were more likely to be acceptors of the COVID-19 vaccine. Among the doers, 19 (8 males and 11 females [42.2%]) had received at least one dose of the COVID-19 vaccine.

**Table 2.** Sociodemographic characteristics of participants

Characteristics	Doers (n=45)	Non-doers (n=45)
Age group (years), n (%)		
18–25	14 (31.1)	23 (51.1)
26–30	10 (22.2)	5 (11.1)
31–35	5 (11.1)	2 (4.4)
36–40	3 (6.7)	8 (17.8)
40–45	2 (4.4)	2 (4.4)
>45	11 (24.4)	5 (11.1)
Sex, n (%)		
Male	25 (55.6)	18 (40.0)
Female	20 (44.4)	27 (60.0)
Level of education, n (%)		
None	4 (8.9)	9 (20.0)
Primary	6 (13.3)	16 (35.6)
Secondary	28 (62.2)	17 (37.8)
Post-secondary	7 (15.6)	3 (6.7)

### Behavioural associations

The differences in the most actionable points outlined in Table 1 that could influence vaccine acceptance behaviour are presented. Only the domains that had at least one significant difference in the response among doers and non-doers are presented, with full details of the comparisons in Supplementary Data File S1.

**Table 3.** Perceived social norms

Determinant	Doers, n (%)	Non-doers, n (%)	Difference (%)	OR	95% CI
Proportion of people you know who will receive the vaccine?					
Don't know/won't say	8 (18)	16 (36)	−18	0.39	0.15–1.04
Who would approve of you receiving the vaccine?					
No one except God	0 (0)	6 (13)	−13	0	0
Who would disapprove of you receiving the vaccine?					
No one will disapprove	23 (51)	12 (27)	24	2.88	1.19–6.95
Don't know	1 (2)	8 (18)	−16	0.11	0.01–0.88
Will most of your close family friends want you to receive the vaccine?					
Yes	38 (84)	13 (29)	56	13.3	4.76–37.5
No	3 (7)	17 (38)	−31	0.12	0.03–0.44
Don't know/won't say	4 (9)	15 (33)	−24	0.20	0.06–0.65
Will most of your community or religious leaders want you to receive the vaccine?					
Yes	33 (73)	18 (40)	33	4.13	1.69 –10.05
No	3 (7)	10 (22)	−16	0.25	0.06–0.98
Will you receive the vaccine if a nurse or doctor recommends it?					
Very likely	25 (56)	7 (16)	40	6.79	2.50–18.41
Not likely	5 (11)	20 (44)	−33	0.16	0.05–0.47

CI: confidence interval.

## Perceived consequences, safety, trust and action efficacy of the COVID-19 vaccine

Table 4 shows differences in responses between doers and non-doers regarding perceived consequences, safety, trust and action efficacy of the vaccine.

**Table 4.** Perceived consequences, safety, trust and action efficacy of the COVID-19 vaccine

Determinant	Doers, n (%)	Non-doers, n (%)	Difference (%)	OR	95% CI
Perceived positive consequences (advantages)					
Protection from contracting the disease	36 (80)	13 (29)	51	9.85	3.72–26.08
No advantages	3 (7)	13 (29)	−22	0.18	0.05–0.67
Don't know	6 (13)	18 (40)	−27	0.23	0.08–0.66
How safe would it be for you to receive the COVID-19 vaccine?					
Not safe at all	2 (4)	17 (38)	−33	0.08	0.02–0.36
Mostly safe	21 (47)	8 (18)	29	4.05	1.55–10.60
Very safe	20 (44)	2 (4)	40	17.20	3.71–79.82
Don't know/won't say	2 (4)	18 (40)	−36	0.07	0.01–0.32
How much would you trust a COVID-19 vaccine?					
No trust at all	4 (9)	23 (51)	−42	0.09	0.03–0.03
Trust it a lot	18 (40)	2 (4)	36	14.33	3.08–66.73
Somewhat likely	17 (38)	8 (18)	20	2.81	1.06–7.43
Not likely at all	13 (29)	5 (11)	18	3.25	1.05–10.07
Don't know/won't say	8 (18)	23 (51)	−33	0.21	0.08–0.54
If one has been infected with COVID-19, the vaccination is not necessary					
Disagree a lot	18 (40)	6 (13)	27	4.33	1.52–12.34
Don't know/won't say	3 (7)	11 (24)	−18	0.22	0.06–0.86
Most people will eventually get infected with COVID-19, so receiving the vaccine is unnecessary					
Agree a little	13 (29)	4 (9)	20	4.16	1.24–14.00
Agree a lot	3 (7)	14 (31)	−9	0.16	0.04–0.60
Disagree a lot	4 (9)	13 (29)	−20	0.24	0.07–0.81

CI: confidence interval.

### Perceived social norms

Table 3 describes the significantly different social norms between doers and non-doers. The greatest difference between doers and non-doers was the perceived approval from friends to receive the vaccine, which was 10.3 times greater among doers than non-doers. Also, doers were 5.1 times more likely to respond that they would receive the vaccine if recommended by a doctor or nurse. Non-doers responded that it was only God's approval that would persuade them to receive the vaccine. The only positive consequence of getting vaccinated perceived by doers was the protection it provided from contracting the disease. There was no significant difference between the two groups in the negative consequences or disadvantages of the vaccine (Supplementary Table S1), however, non-doers were 11.3 times more likely to feel the COVID-19 vaccine was not safe at all. Non-doers lacked trust in the COVID-19 vaccine and were 12 times more likely to report ‘no trust at all’ compared with doers.

### Perceived self-efficacy and access to the COVID-19 vaccine

Table 5 describes the factors that would facilitate or hinder receiving the COVID-19 vaccine. Decentralization of vaccination services within the neighbourhood or door-to-door administration were factors that doers felt would facilitate vaccine uptake. Doers were also more likely to report that a stressful vaccination process would hinder uptake. Non-doers were 19.1 times more likely compared with doers to report that it was ‘very difficult’ to get to the vaccination centre, while doers reported 2.8 more times that it was ‘not difficult at all’.

**Table 5.** Perceived self-efficacy and access to COVID-19 vaccine

Determinant	Doers, n (%)	Non-doers, n (%)	Difference (%)	OR	95% CI
What would make it easier to receive the vaccine?					
Decentralizing vaccination to streets or door to door	17 (38)	5 (11)	27	4.86	1.60–14.71
Nothing will make it easier	11 (24)	26 (58)	−33	0.24	0.10–0.58
What would make it difficult to receive the vaccine?					
If getting the vaccine is stressful	8 (18)	1 (2)	16	9.51	1.14–79.61
Don't know	3 (7)	17 (38)	−31	0.12	0.03–0.44
How difficult is it to get to the centre where vaccines are administered?					
Very difficult	1 (2)	15 (33)	−31	0.05	0.01–0.36
Not difficult at all	23 (53)	12 (27)	27	3.14	1.30–7.60

CI: confidence interval.

### Perceived susceptibility to COVID-19 infection and the role of divine will and information towards receiving the vaccine

Table 6 shows the differences between doers and non-doers regarding susceptibility to infection and the role of divine will and information. Doers were more likely to believe in being susceptible to the virus and in God's approval of the vaccine. Information provided by religious leaders was considered trustworthy, particularly among non-doers.

**Table 6.** Perceived susceptibility to COVID-19 infection and the role of divine will and information towards receiving the vaccine

Determinant	Doers n (%)	Non-doers n (%)	Diff.* (%)	Odds ratio	95% CI
Likelihood of someone in your household getting COVID-19 infection in the following 3 months?					
Very likely	10 (22)	3 (7)	16	4.00	1.02–15.68
Does God approve or disapprove of people receiving the COVID-19 vaccine?					
God approves	22 (49)	11 (24)	24	2.96	1.21–7.25
God does not approve or disapprove	5 (11)	18 (40)	−29	0.19	0.06–0.57
Don't know/won't say	6 (13)	18 (40)	−27	0.23	0.08–0.66
Would you trust the information religious leaders provide on the safety and efficacy of the COVID-19 vaccine?					
Very high level of trust	2 (4)	9 (20)	−16	0.19	0.04–0.92
Have you seen anything or heard rumours that would stop you from receiving the COVID-19 vaccine?					
No	30 (67)	20 (44)	22	2.50	1.06–5.87

CI: confidence interval.

## Discussion

The main findings from this study are that perceived social norms (approval from family, friends and health workers) and positive consequences (protection) among doers were determinants of behaviour. Negative consequences, perceived poor access, safety concerns, lack of trust, low vaccine efficacy and low susceptibility among non-doers were among the most important determinants of behaviour. Non-doers were less likely to provide reasons for their behaviour across all aspects of the inquiry, implying that their beliefs are less likely evidence-based but may be related to widespread conspiracy theories that have contributed to COVID-19 hesitancy and low uptake globally.^15^

Perceived poor access had the strongest association with being a non-doer in this study, aligning with the theory of planned behaviour whereby perceived behavioural control bears strongly on the intention to adopt a behaviour.^16^ In the Makoko community, COVID-19 vaccines are available free of charge at the primary healthcare centre, but accessing this service may be challenging, particularly for those living on the water. This implies that low vaccine uptake in Makoko may transcend vaccine hesitancy, as access may be an additional challenge despite availability. The doers in this study recognized that a stressful vaccination process would limit vaccine uptake and, based on this and previous reports, decentralization of vaccination sites is a potential strategy for improving vaccine uptake.^13^ Successful vaccination drives that brought the vaccine close to the people in other low-income communities underscore the veracity of this intervention.^17^ Also, a previous study in Nigeria suggested that logistical challenges such as transportation costs and financial loss due to time spent away from work may have the greatest impact on COVID-19 vaccine uptake.^18^ As recommended by the participants in this study, door-to-door vaccination campaigns in Makoko, especially for those living on the water, could remove an important barrier to vaccine uptake.

Perceived social norms have remained consistent as a determinant of COVID-19 vaccine acceptance, both in this study and in previous studies.^5,13,18^ The greatest influence among doers in our study was the opinion of family and friends, followed by the recommendation of a healthcare worker. For non-doers, they believed that only God could make them take the vaccine, indicating a strong conviction to reject the vaccine. The importance is that while social norms could encourage vaccine uptake, changing the behaviour of those who already have negative conceptions may require a different dimension of engagement. For example, trusted persons such as religious leaders and healthcare workers may have a greater impact on the delivery of COVID-19 vaccine education and the role of community leaders may be limited.^13,18,19^ Community engagement strategies that have leveraged the trust in healthcare workers and religious leaders have been shown to be effective in marginalized communities, particularly when messaging is factual and delivered with empathy and respect.^20^ Educating adolescents and young adults in schools to serve as role models and advisers for older adults is another strategy. Development of the messaging for this community must also take into account the fact that the population may perceive innate protection based on strong spiritual beliefs and long-standing customs, making the expected protection from the vaccine less convincing.^13,18,21^

According to the health belief model, behaviour is influenced by perceived susceptibility to the disease, the benefits of intervention and the ability to act to mitigate it.^22^ Aspects of vaccine hesitancy in Makoko are driven by beliefs of low susceptibility to the infection among non-doers. Narratives of interviews conducted among community leaders demonstrate that acquiring the disease was not a priority. Rather, the lack of food, healthcare services and other social amenities was a greater concern.^21^ Therefore, efforts to improve the COVID-19 vaccine uptake in low-income settings such as Makoko must incorporate programs that improve social welfare, including the provision of other basic healthcare services such as blood pressure measurements and other routine health checks.

Perceived low effectiveness of the COVID-19 vaccine among non-doers reflects the low level of knowledge.^13,17,18^ Although dosing for the COVID-19 vaccine is evolving, the benefits of primary vaccination (one or two doses depending on vaccine type) against serious disease and mortality, even from the new variants, are recognized and this information must be highlighted in the messaging.^23^ Experiences of unvaccinated community members who recovered from severe COVID-19 and those who have received the vaccine could be shared first hand during community programs as evidence of the potential severity of the disease and safety of the vaccine. Conspiracy theories are widespread globally and have been linked with vaccine hesitancy. However, these were not proffered in this study and must be acknowledged and debunked as part of the educational activities.^15^

Concerns about vaccine safety are valid, but they are also fuelled in part by misinformation. In a systematic review of confidence and acceptance of the COVID-19 vaccine, concerns about side effects and safety were the most dominant reasons for hesitancy.^5^ However, COVID-19 vaccines are safe and effective and the risk of serious adverse effects for the nearly 10 billion doses administered worldwide remains <1%.^24^ In Canada, 95% of reported adverse effects were considered not serious and the WHO concludes that the benefits of vaccination far outweigh the potential risk of serious side effects.^25,26^ Therefore, information about the safety and side effects of the vaccine must be well constructed and delivered to reduce apprehension. Mild side effects such as headache, malaise, pains and transient fever, which are common across multiple vaccine types, should be distinguished from serious adverse effects.^24^

A recognized limitation in this study partly lies in those of the BA approach, which relies mainly on behavioural change through reflective motivation, with less emphasis on the availability of resources and infrastructure. In this study, despite the availability of the vaccine free of charge to the community, access may be limited due to geographic and infrastructure challenges, particularly for the communities on the water, as alluded to by some non-doers. Future studies are needed to untangle the issue of access for a better assessment of vaccine hesitancy in this community. In the meantime, the vaccination of adults for COVID-19 could be incorporated into the monthly door-to-door vaccination drives used to administer routine childhood immunizations in the Makoko water communities. Furthermore, the BA approach does not assess the impact of sociodemographic factors, including gendered issues on health decision making. The attitudes towards the COVID-19 vaccine may be more nuanced beyond doers and non-doers, hence those who may be hesitant or undecided would have been missed or forced into one of the two categories. We are also aware that the conduct of this study in a unique urban slum in Lagos limits the generalizability of our findings. However, despite these limitations, we conducted a rapid assessment of a unique urban slum in Lagos and provided insights and direction for timely interventions to enhance COVID-19 vaccine uptake.

## Conclusions

This study provides additional data on determinants of COVID-19 vaccine acceptance from a LMIC. We used the BA approach, which allows for rapid assessment of behaviour and the timely development of interventions. We focused on one of the most marginalized urban slums globally and report that perceived inaccessibility, low efficacy, safety concerns and mistrust were major drivers of non-acceptance. We deduce that measures to improve COVID-19 vaccine acceptance must address issues related to accessibility and misinformation through the decentralization of vaccination sites and by providing clear, concise and evidence-based education on vaccine efficacy and safety. Community education should leverage the trust in healthcare workers and religious leaders by the community and on the testimonials of individuals with experience with the vaccine and/or the disease. Improvements in social welfare should be linked to COVID-19 vaccine uptake campaigns considering the valid concerns of meeting basic needs that make vaccination less of a priority. Social mapping is also needed to understand the areas of greatest need and to provide guidance for health promotion that builds and maintains trust in the vaccine.

## Data Availability

The data supporting the findings of this study are available from the corresponding author upon reasonable request.
